# Grand challenges in physical neuroergonomics

**DOI:** 10.3389/fnrgo.2023.1137854

**Published:** 2023-02-16

**Authors:** Stéphane Perrey

**Affiliations:** EuroMov Digital Health in Motion, Univ Montpellier, IMT Mines Ales, Montpellier, France

**Keywords:** muscle, monitoring, multimodal, neuromechanics, physiology, mobile brain imaging, workload, fatigue

## 1. Introduction

When studying the sensorimotor performance of the brain at work (i.e., the core of neuroergonomics), it is essential to consider the engines of physical work –the muscles– specifically at the behavioral level. The main functions of the human musculoskeletal system are to sustain and modulate force production and provide suitable movement with efficacy. Movement is the most common final output of nervous system activity. Executive behaviors range from whole-body movement, including locomotion, to skilled movement sequences of body parts in realistic environments for workers, such as surgeons performing an operation or sportsmen skiing, shooting, or putting a ball in a hole. French scientist Marey (1894) defined human locomotion in 1894 as a series of physiological actions that are created by human as a whole to move in space in a series of successive moments. His research allowed the establishment of the first rules governing the maneuvers of soldiers, features of physical exercises, and operations carried out by workers. To do so, he used some behavioral methods already belonging to physical neuroergonomics. As outlined in Dehais et al. (2020), the section Physical Neuroergonomics of the journal Frontiers in Neuroergonomics is concerned with “the human brain in control of muscular performance, movement, and brain-body interrelationships in conditions of health, workplace, fatigue, training, injury, and disease states”. Thus, physical neuroergonomics “focuses on human physical capabilities and limitations, pertaining to neuro/physiology and biomechanics responses of the human body, as they relate to physical work” (Karwowski et al., 2003).

**Figure 1:**
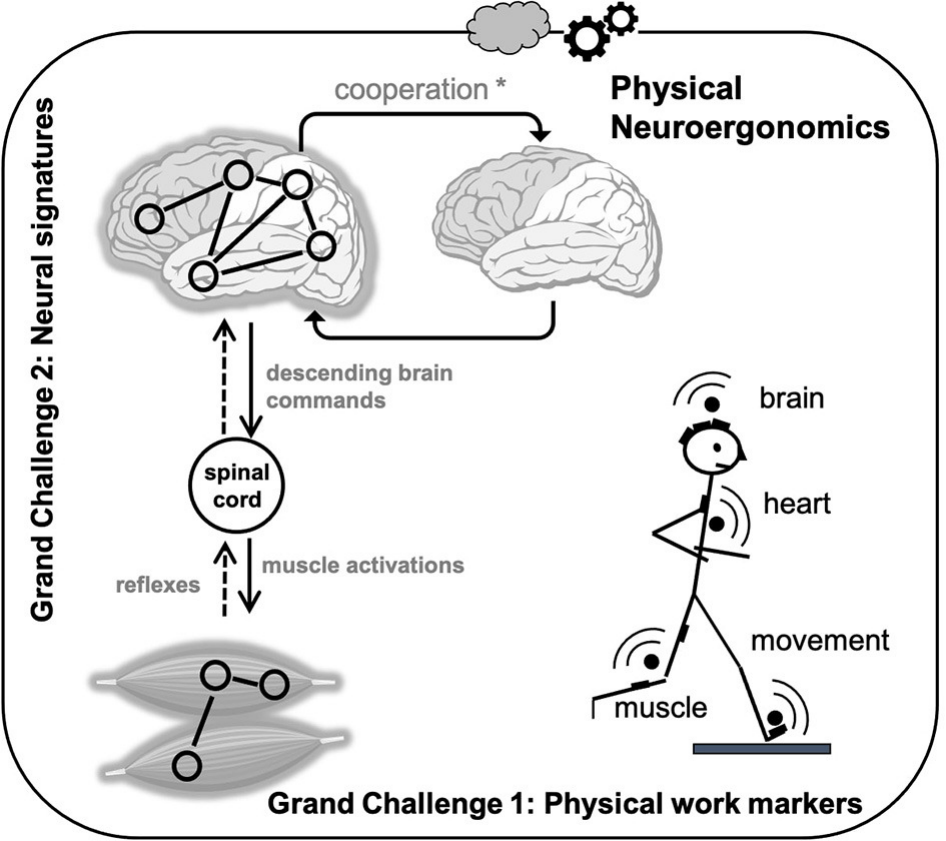
Illustration of the two main grand challenges for Physical Neuroergonomics where a multimodal approach is needed for incorporating different sources of information to allow a robust extraction of physical work features. Regarding the grand challenge 1, heart monitoring is used along with other wearable sensors like accelerometers, power meters, etc. Heart rate has long been acknowledged as a key indicator of physiological adaptation, exercise intensity, and workload effort at a central level. However, another stream of physical neuroergonomic markers can depend on the electrical and physiological dynamics demands at a more local site, giving complementary and precise signatures of muscle or cerebral activities. Regarding the grand challenge 2, in the process of human voluntary movement, synergy occurs between different regions of the brain, between different muscles, and between the cortex and muscle reflecting the information flow within a cortico-peripheral-cortical loop, involving both descending and ascending (reflexes) pathways. Information flow also occurs between interacting human brains during a cooperative task. ^*^Note that the combination of behavioral, muscle, and brain data might be used to identify cooperative interaction among individuals.

Generation and control of force are required to walk, manipulate objects, or play sports. On one hand, the ability to accurately produce the various ranges in force with good timing can be accomplished through modifying the firing properties and recruitment order of motor units. In order to generate force voluntarily, on the other hand, the sensorimotor network, a large-scale brain network, must be able to communicate efficiently with the motor neurons in the spinal cord which are responsible for force generation through muscle contraction (Figure 1). Within this basic framework, the neuromuscular control system exhibits tremendous flexibility and adaptability in response to various (loco)motor tasks, different physical work parameters (e.g., force, load, and velocity), acute muscular fatigue, and detraining, injuries, and diseases. When sharing load among multiple muscles, or even with other people, humans are able to select an optimal pattern of muscle activation that minimizes costs from an optimal control theory perspective (Harris and Wolpert, 1998). Also, the human body has many degrees of freedom to generate basic movements, such as reaching or grasping. Nikolai Bernstein first proposed the idea of muscle synergies to explain how the nervous system simplifies control of a vast number of independent parameters (Bernstein, 1967). Each muscle synergy generates a particular mechanical action, and the flexible combination of muscle synergies is thought to allow a wide repertoire of natural motor behaviors.

Despite its great importance in physical neuroergonomics, how the peripheral and central nervous systems achieve optimal behavior and “cooperate” in actual field/work settings remains poorly understood, particularly within the context of multiple and repetitive operating actions when muscle output performance is challenging. During sustained or repeated muscle contractions, muscle force declines throughout time at a specific rate. This transient loss of work capacity resulting from preceding workload, otherwise called fatigue, is one of the most fundamental biological topics both for research and practical application in workplace, sport, and health domains. Fatigue limits human performance in normal conditions and even more so in disease. Task-induced (neuro)muscular fatigue (i.e., motor performance fatigue) is considered in healthy people as a reversible loss of muscle force during work over time, which occurs as a safety mechanism. The central nervous system is thought to play a ‘protecting’ role to prevent injury or damage by proposing different strategies. Inadequate rest periods do not allow time for proper recovery, which could increase the chance of injury and decrease the productivity of workers, such as athletes. Many injuries in the workplace and in sports are in this way caused by overuse. Using a range of outcome measures in order to achieve a thorough understanding of which factors contribute to force-generation capability of the muscles according to the environment is needed in physical neuroergonomics. This will provide new avenues for developing adapted countermeasures and workplace environments. One potential application of physical neuroergonomics could be to inform the design of workstations and user interfaces (e.g. cockpits) for more intuitive interaction and to reduce fatigue. From this, two grand challenges, illustrated in Figure 1, are discussed in the next sections.

## 2. Grand challenge 1: Identifying, monitoring, and classifying the markers of physical workloads

Most people are exposed to demanding workloads during physical tasks that can increase injury, illness risk, musculoskeletal disorders, and cause fatigue. Synergistic multimodal approaches to biomarkers analysis in physical work, profiling, and monitoring can offer an opportunity to gain insight into an individual's biomechanical and neurophysiological status. When combined with other contextual information, these approaches provide evidence-based guidelines and strategies to reduce the risk of musculoskeletal disorders and injury in humans, while also optimizing their whole performance. In this regard, objective, accurate, repeatable, and relevant simultaneous measurements of the muscle and brain functions should make it possible to assess the effectiveness of the work capacity, follow its progress, or to adapt some specific needs for users. In physical neuroergonomics, one main challenge is centered on the ability of the musculoskeletal, cardiopulmonary, and central nervous systems' responses of people to be assessed and quantified in a non- invasive and unobtrusive manner to diagnose muscle and motor performance, and to restore specific functions due to fatigability, injury, or disease. A multivariate approach at a multidimensional, neuro-mecano-physiological level to monitor and quantify task loads in the workplace is thus highly required. This first technological and methodological challenge is fundamentally one important to physical neuroergonomics. Built on new emerging wearable technologies allowing real-time monitoring, a new neuroergonomics-type framework can be proposed in which neuro- physiological and mechanical adaptation pathways are considered altogether.

Connected wearable technologies allow for continuous monitoring of human physiology and movements during our daily activities and living. In sports, these markers have found broad utility in quantifying the training load and modeling physical performance of athletes (Imbach et al., 2022). In the workplace, characterization of the association between working postures and work-related musculoskeletal disorders requires accurate posture measurement for estimating occupational exposure to physical risk factors. The external load/demand supported by one person is often considered as the total (loco)motor and mechanical stress produced by physical work. The so-called external load, defined as the work completed, can include measures of velocity of movement, distance covered, number of repetitions, magnitude of impacts, acceleration number, and work-specific movements such as reaching and grasping. In many situations, repetitive external loads are imposed on the human body due to its interaction with the environment. Ground reaction force is a relevant indicator of the external force and mechanical effort imposed during locomotion tasks and has been considered the main representative and most adequate measure of the impact received by the lower-extremity musculoskeletal system (Zadpoor and Nikooyan, 2011). Motion capture systems are the most used methods to measure external load based on robust biomechanics knowledge. In the health science industry, precise, traditional motion capture is heavily constrained by complex sensors and acquisition environment. To address these limitations, marker-less techniques have to be prioritized and are now accessible (Nakano et al., 2020).

There is an emerging interest in using sensors to capture fine-grained physical behaviors in various domains of daily life over long duration and/or requiring motor skills (open skills sport, surgery, engineering activities, etc.). With increasingly available wearable technologies, activity trackers with 3-axial accelerometers used to assess physical work unobtrusively, objectively, continuously, and routinely can capture daily activity occurring in real life to improve physical activity and related physiological (Ferguson et al., 2022) and sports performance (Imbach et al., 2020) outcomes. However, a high degree of instrumentation (many IMUs at different body positions) is not always acceptable in a sports or company context since it would hinder mobility. In a real work setting, examining the accuracy performance of the IMU sensors (even holding with suits) during the completion of multiple work tasks with different relevant kinematic characteristics is still necessary. By combining wearable sensors and powerful pattern recognition techniques, an automatic activity recognition system for workload monitoring in users could be established (Zhang et al., 2022). Deep learning approaches have not yet fully reached the field of sensor-based work activity recognition despite its successes in image and speech recognition.

Finally, monitoring and quantifying human-body kinematics during real situations for an extended duration could require alternative technologies in a more non-invasive and accurate manner. Skin-interfaced wearable devices can monitor the stresses on muscles and joints and offer highly localized motion capture capabilities from multimodal sensors in demanding environments (Ray et al., 2019). Muscle performance relies not only on biomechanics markers; understanding the underlying physiology of motor tasks while operating in environments allows the personalization of physical work activities, as well as the identification of potential health risks and maladaptation. In that direction, electromyographic (EMG) measurements by soft, stretchable electrode arrays interfaced to the skin can record the electrical signal associated with muscle activation. Changes in oxygen levels can provide complementary insights into the function of the skeletal muscle in both healthy and diseased states. Near-infrared-spectroscopy (NIRS) can study the dynamics of oxygen levels in skeletal muscle during physical tasks (Perrey, 2022). Combined EMG and NIRS offers a more complete window into neuromuscular functioning that can be collected over a long time period. Recent developments in miniaturizing and embedding the devices in clothing garments (Giminiani et al., 2020) suggest that this modality of localized demand quantification not only has merit but also could have extended applications in the near future for physical neuroergonomics.

Importantly, physical neuroergonomics has to be associated with mental demand and the related fatigue. In the real world, the amount of mental resources employed by the individual increases to deal with the task difficulty demand (i.e., the mental workload), especially when performing complex movement. Dual-task studies of simultaneous exercise and cognitive tasks demonstrated that the stability and accuracy of cognitively demanding motor tasks is affected (Mandrick et al., 2013). Concurrent demanding physical and mental activities may exacerbate motor performance fatigue (Srinivasan et al., 2016). When physical demand imposes constraints that may increase mental demand or increase the difficulty of the motor task, individuals may have less capacity to handle these constraints, resulting in movement patterns that may induce subsequent injury (Burcal et al., 2019). Thus, subtle neuro- mechanical and physiological information from wearable sensors combined with advanced machine learning methods can serve as the basis of monitoring mental and physical workloads and classification in physical neuroergonomics.

## 3. Grand challenge 2: Revealing the multiple neural signatures to physical workloads

Physical neuroergonomics aims to understand the neural activities related to human performance in real-world task settings, meaning that it involves the investigation of several neural systems interacting together during real-world conditions. While a substantial role of neural activities in controlling muscle performance in laboratory settings has been identified, a lack of research in naturalistic settings remains. In addition, most physical activities studied in a laboratory setting are isometric, finger movements, as well as basic whole-body movement (e.g., cycling) mimicked on a dedicated ergometer. Thus, the second grand challenge is related to how we reveal and discriminate the interaction dynamics either at the muscle or the cortex levels, as well as between the brain and muscle, to achieve individual muscle performance outputs on the field and during cooperative tasks of two or more persons simultaneously. Although changes in motor unit recruitment mediated by central mechanisms can be indirectly reflected in EMG signals, it is difficult to analyze the neural mechanisms behind muscle state at a systemic level using the single EMG-based method. Rather than focusing on isolated muscles or cortical areas, functional networks should be constructed at both the muscle and cerebral levels (Liang et al., 2021). It will relay how the central nervous system coordinates the activity of multiple muscles to achieve a variety of behavioral goals, and addresses the interaction among the involved sub-systems (muscles, joints, nervous system), instead of analyzing the response of each sub-system separately. In addition, hyperscanning paradigms (Liu et al., 2018) provide a valuable platform for observation of neural signatures of cooperative motor actions from more than one individual in the real world at the peripheral and central levels. We propose four levels of investigation in studying functional networks.

Constructing muscle networks to reveal different connection patterns of synergistic and antagonistic muscles has become an emerging method to explore the neuromuscular control mechanism (Boonstra et al., 2015; Kerkman et al., 2020). Muscle network analysis quantifies the functional connectivity between motion-related muscles and is able to identify the frequency characteristics of specific muscles (De Vries et al., 2016) that are regulated by common neural inputs. Therefore, muscular network analysis can reveal the characteristics of functional separation and integration for the neuromuscular system, which is suitable for analyzing the neuromuscular control mechanisms behind muscle state fatigue. With high-density EMG based muscle synergy, it permits more spatial resolution in the recording of muscle activities, so that it may be possible to isolate activities of different within-muscle compartments that are under different controls of the central nervous system (Geng et al., 2022). Finally, a new conceptual framework of the neural control of movement can be considered by merging the concept of common input to motor neurons and modular control, together with the constraints imposed by recruitment order (Hug et al., 2023).

During physical activity, not only do the neurophysiological changes play a role at a peripheral level, but brain activities among interconnected regions also vary based on the type of motor tasks in relation to the force, the movement velocity, and amplitude. In addition, brain areas associated with attention, motor planning, sensorimotor, and cognitive processing at different brain regions (frontal, central, and parietal) are altered during tasks that require a high level of physical exertion. These adaptive changes occurring during a physical task emphasize the need for identifying the involved neural systems. Several advanced methods for deriving relevant information regarding neural fingerprints at multiple scales should be implemented in physical neuroergonomics. Discrimination between different levels of intensities or exertion during physical work could be reflected in the functional connectivity of the brain network, mainly in the prefrontal motor and central areas. Further insight into the physical work-induced functional changes in brain activation patterns should be explored by mapping cortical networks during physical tasks rather than assessing localized brain activation. Functional brain connectivity has the potential to provide a more global picture of the continuous temporal evolution of brain activity regarding various physical scenarios. It allows for detection of physical activity-dependent network reorganization. Several modeling approaches of functional brain connectivity based on EEG (Ismail and Karwowski, 2020) and fNIRS (Vergotte et al., 2018) during physical tasks have been proposed and should benefit the physical neuroergonomics field.

On the other hand, it has been reported that the coupling relationship between scalp EEG and EMG in a specific frequency band can reflect the functional coupling between the motor cortex and working muscles (Mehrkanoon et al., 2014). The role of this functional corticomuscular coupling in different frequency bands during various physical tasks might provide important information to further reveal the underlying neural mechanisms of muscle state in health and disease (Cremoux et al., 2017; Fauvet et al., 2021).

Finally, shared brain dynamics assessed with EEG or fNIRS during joint tasks can be revealed in multiple interacting people (Babiloni and Astolfi, 2014). The study of the neurophysiological bases of cooperative behavior is crucial for all of the applications in which strict collaboration between two or more people is mandatory for the success of the task. Functional brain connectivity analyses in examining relationships across activated regions provide insight on how individuals may process and interpret information, engage with the task, and their potential impact on motor functional outcomes. Considering the existing body of hyperscanning literature, a current challenge in physical neuroergonomics that needs to be addressed is studying complex motor behaviors during social interactions occurring in a natural environment between two or more individuals. For that purpose, inter-brain synchronization (EEG-fNIRS) with measures at the cortico-spinal level (EMG hyperscanning) may be used to further characterize the neural dynamics of physical interactions between individuals.

## 4. Conclusion

In an effort to defy simple reductionist explanations, new appealing inter-disciplinary research areas are necessary for a better understanding of the amazing ability of the brain to reorganize in response to muscle contractions brought on by various physical work. Physical neuroergonomics can provide some potentially fruitful new directions for combining research in biomechanics, neurophysiology, physiology, sport sciences, data science, neuroimaging, and neurosciences reflecting physical processes. The first grand challenge is focused on the use of small, ubiquitous sensors and/or innovative methods for performance tracking of brain-muscle relationships across a variety of physical domains by taking advantages of state-of-the-art data science methods. The second grand challenge is related to the development of methods capable of building an integrated picture of the multi-scale functional networks within the muscle, the brain, and between the brain and muscle. All in all, it will have a marked impact on our understanding of limiting factors of the physical work in healthy and diseased individuals and in evaluating their relationship with whole performance, injury risk, and safety concerns. The phrase “The whole is more than the sum of its parts” aptly defines the modern concept of synergy visible for each of the sub-challenges of the section Physical Neuroergonomics.

## Author contributions

SP contributed to the manuscript draft, revision, read, and approved the final submitted version.
